# Scenario‐free robust optimization algorithm for IMRT and IMPT treatment planning

**DOI:** 10.1002/mp.17905

**Published:** 2025-05-25

**Authors:** Remo Cristoforetti, Jennifer Josephine Hardt, Niklas Wahl

**Affiliations:** ^1^ Department of Medical Physics in Radiation Oncology German Cancer Research Center – DKFZ Heidelberg Germany; ^2^ Heidelberg Institute for Radiation Oncology – HIRO Heidelberg Germany; ^3^ Faculty of Physics and Astronomy Heidelberg University Heidelberg Germany

**Keywords:** 4D robust optimization, robustness, treatment planning

## Abstract

**Background:**

Robust treatment planning algorithms for intensity modulated proton therapy (IMPT) and intensity modulated radiation therapy (IMRT) allow for uncertainty reduction in the delivered dose distributions through explicit inclusion of error scenarios. Due to the curse of dimensionality, application of such algorithms can easily become computationally prohibitive.

**Purpose:**

This work proposes a scenario‐free probabilistic robust optimization algorithm that overcomes both the runtime and memory limitations typical of traditional robustness algorithms.

**Methods:**

The scenario‐free approach minimizes cost‐functions evaluated on expected‐dose distributions and total variance. Calculation of these quantities relies on precomputed expected‐dose‐influence and total‐variance‐influence matrices, such that no scenarios need to be stored for optimization. The algorithm is developed within matRad and tested in several optimization configurations for photon and proton irradiation plans. A traditional robust optimization algorithm and a margin‐based approach are used as a reference to benchmark the performance of the scenario‐free algorithm in terms of plan quality, robustness, and computational workload.

**Results:**

The implemented scenario‐free approach achieves plan quality similar to traditional robust optimization algorithms, and it reduces the distribution of standard deviation within selected structures when variance reduction objectives are defined. Avoiding the storage of individual scenario information allows for the solution of treatment plan optimization problems, including an arbitrary number of error scenarios. The observed computational time required for optimization is close to a nominal, non‐robust algorithm and substantially lower compared to the traditional robust approach. Estimated gains in relative runtime range from approximately 5–600 times with respect to the traditional approach.

**Conclusion:**

This work introduces a novel scenario‐free optimization approach relying on the precomputation of probabilistic quantities while preserving compatibility with state‐of‐the‐art uncertainty modeling. The measured runtime and memory footprint are independent of the number of included error scenarios and similar to those of non‐robust margin‐based optimization algorithms, while achieving the required dose and robustness specifications under multiple different optimization conditions. These properties make the scenario‐free approach suitable and beneficial for 3D and 4D robust optimization involving a high number of error scenarios and/or CT phases.

## 1 INTRODUCTION

The effective handling of uncertainties, such as setup and range uncertainty as well as anatomical changes, poses a fundamental challenge for external beam radiotherapy treatment planning.^1^ Nevertheless, modeling these multiple sources of uncertainty and implementing feasible robust optimization strategies is beneficial to preserve plan quality in case of errors manifesting during treatment.^2^

Different treatment modalities, tumor sites, and patient anatomy require different approaches to handling uncertainties during computational treatment planning when they can not be mitigated in preparation and setup.^2^, ^3^, ^4^, ^5^, ^6^, ^7^ When focusing on treatment plan optimization, the gold standard approach to ensure target coverage relies on the definition of planning margins. This approach has proven reliable under the assumption that the static dose cloud approximation holds and is particularly effective for intensity modulated radiation therapy (IMRT).^8^, ^9^

The static dose cloud approximation assumes that rigid shifts of the dose distribution well approximate the impact of main sources of uncertainties, such as set up errors or organ motion. It is generally only valid for regions of homogeneous density and target sites of reproducible positioning.^10^ Such assumption cannot generally be extended to the case of protons and heavy charged particle irradiation, given the high sensitivity to range uncertainty.^5^, ^6^

For cases in which the static dose cloud approximation breaks down, robust optimization algorithms were developed to explicitly include a representation of the uncertainty model into the optimization problem.^2^, ^7^ The scenario‐based representation is a generalization of the margin‐concept for the non‐static case^10^, ^11^ and thus bypasses the definition of planning margins with the optimization focusing on the clinical target volume (CTV).

Robust optimization is particularly beneficial in highly non‐static treatment configurations such as lung cancer irradiation.^12^, ^13^ Additional uncertainty introduced by the breathing motion can be accounted for by including 4D‐CT datasets, thus performing a 4D‐robust optimization. Use of 4D‐robust optimization can improve target coverage and OAR sparing as opposed to non‐robust optimization based on margin definition.^14^

Different mathematical formulations of the optimization problem can be exploited to achieve robustness goals,^7^, ^15^, ^16^, ^17^ however, the computational implementation of such algorithms generally relies on, and is burdened by, the estimation of multiple error scenario distributions.^18^ A statistically stable representation of the uncertainty model requires a sufficiently large amount of error scenarios to be sampled from the underlying probability distribution.^16^, ^19^ Therefore, such algorithms always imply a trade‐off between efficacy and feasibility.

Typical examples of robust optimization algorithms are the *minMax* ^20^ and the *stochastic*,^21^ sometimes also called *expected value*, approaches.^2^, ^7^ These algorithms have proven to be more effective with respect to margin based approaches in achieving dose conformity and reducing the impact of uncertainties on the optimized distributions.^22^, ^23^ At the same time, they bear intrinsic applicability limitations due to high memory and computational time demand.

Optimization time constraints are of particular concern, especially when 4D‐robust optimization is applied.^24^

The present work explores the capabilities of an alternative, “scenario‐free” robust optimization algorithm. With this approach, the optimization problem's design does not rely anymore on the explicit evaluation of individual scenario distributions. The approach is derived from previous works on probabilistic dose calculation and optimization^15^, ^16^, ^19^ and the expected value approach. It relies on precomputation of expected dose and variance influence terms and thus does not require the storage and computation of multiple scenarios during optimization. Dosimetric objectives are therein defined on the expectation value of the dose, while variance‐reduction objectives are applied to each structure to minimize the mean variance value.

The key advantage of our approach is that the dimensionality of the pre‐computed expected dose and variance influence objects is independent of the number of scenarios (in the case scenarios are used for their construction). This way, the optimization becomes scenario‐free and the approach addresses simultaneously the memory limitation issue and the increased computational time demand of iterating through scenarios during each iteration in optimization.

The algorithm was tested in several treatment configurations. Results were initially collected for the simplified setup of a cubic, homogeneous phantom and extended to clinically realistic cases of lung cancer patients. In both cases, 4D datasets were included to highlight the feasibility of applying the algorithm to otherwise unfeasible optimization configurations. Comparison of plan quality and robustness metrics was performed with respect to margin‐based and traditional robust optimization algorithms. The considered sources of uncertainty include setup and range uncertainties, while the multiple CT‐phases for the 4D optimization were exploited to model patient motion and anatomical changes.

## 2 MATERIALS AND METHODS

### 2.1 Robust optimization

In its general formulation, the robust treatment planning optimization problem aims at finding optimal beamlet intensities $x^{*}$ by solving

(1) $$\begin{array}{cc} x^{*} & =\text{arg}\;\;\min_{x}\;R_{U}\left[F(x)\right]\\ \text{s.t.}\;x & >0\\ c_{n}\left(x\right) & \leq 0 \end{array}$$

where F denotes the global cost‐function to be minimized depending on the beamlet intensity vector x. $R_{U}$ denotes a robustness operator acting on F depending on an uncertainty model U, and the problem can be subjected to by multiple constraint functions $c_{n}$.

F usually represents a multi‐criteria decision function, for example, via the weighted‐sum scalarization $F=\sum_{i}p_{i}f_{i}$ of multiple objectives $f_{i}$ weighted with $p_{i}$. Most, if not all, of these terms will depend on the dose distribution d(x)=Dx, computed as a linear transformation of x with a precomputed *dose‐influence matrix* D.

As dose is the main quantity affected by the uncertainty model, applying $R_{U}$ usually observes possible variation in d due to the uncertainties U. A common way of doing so consists in sampling an arbitrary amount of *error scenarios* from a probability distribution describing the uncertainty model U. The robust operator R then can take many forms, with the most prominent being *minimax*, *Conditional Value at Risk*, or *expected value*. For the approach presented in this manuscript, let us consider the latter expected value, often also named *probabilistic*, approach of

(2) $$R_{U}\left[F(d(x))\right]=E\left[F(d(x))\right]\approx \sum_{s}\pi_{s}\left[F(d_{s\in S}\left(x\right))\right]\;.$$

Equation (2) approximates the expectation value operator E by evaluation of a set of error scenarios S with importance weights $\pi_{s}$. Note that, in general, evaluation of each dose scenario $d_{s}=D_{s}x$ entails storing a scenario dose‐influence matrix $D_{s}$.

### 2.2 Scenario‐free robust optimization

#### 2.2.1 Reformulation of the probabilistic optimization problem

The developed scenario‐free probabilistic approach is based on previous work by Bangert et al.,^15^ which used a probabilistic dose calculation algorithm to find a fully analytical approximation of the expectation value operator in (2) using a precomputed expected dose influence matrix E[D] and total variance influence matrices Ω.

To do so, Bangert et al.^15^ started from the special case of the penalized least‐squares objective function

(3) $$F\left(d\right)={\left(d-d^{\mathrm{ref}}\right)}^{T}P\left(d-d^{\mathrm{ref}}\right)$$

and its expectation value

(4) $$E\left[F\right]=\mathrm{tr}\left(P\Sigma_{d}\right)+{\left(E\left[d\right]-d^{\mathrm{ref}}\right)}^{T}P\left(E\left[d\right]-d^{\mathrm{ref}}\right)\;.$$

In Equations (3) and (4), $d^{ref}$ is the prescribed dose distribution, and $P=\mathrm{diag}(p_{1},p_{2},\cdots ,p_{N})$ is a diagonal matrix assigning penalty factors $p_{i}$. Equation (4) is composed of two terms; a “total variance” term $\mathrm{tr}(P\Sigma_{d})$ representing the penalty‐weighted sum of variance ($\Sigma_{d}$ is the dose's covariance matrix), and the penalized quadratic difference of the expectation value to the prescription.

Evaluation of both terms can now be facilitated by aforementioned total variance influence Ω and expected dose influence matrix E[D]. While this is trivial for the second term using

(5) E[d]=E[D]x

similarly to nominal dose evaluation, rewriting the variance term requires a closer look using multiple properties of the matrix trace:

(6) $$\begin{array}{cc} \mathrm{tr}(P\Sigma_{d}) & =\mathrm{tr}\left(P(E\left[dd^{T}\right]-E\left[d\right]E{\left[d\right]}^{T})\right)\\ & =\mathrm{tr}\left(P\left(E\left[Dxx^{T}D^{T}\right]-E\left[D\right]xx^{T}E{\left[D\right]}^{T}\right)\right)\\ & =\mathrm{tr}\left(xx^{T}E\left[D^{T}PD\right]-xx^{T}E{\left[D\right]}^{T}PE\left[D\right]\right)\\ & =\mathrm{tr}\left(xx^{T}E\left[D^{T}PD\right]\right)-\mathrm{tr}\left(xx^{T}E{\left[D\right]}^{T}PE\left[D\right]\right)\\ & =x^{T}E\left[D^{T}PD\right]x-x^{T}E{\left[D\right]}^{T}PE\left[D\right]x\\ & =x^{T}\left(E\left[D^{T}PD\right]-E{\left[D\right]}^{T}PE\left[D\right]\right)x\\ & =x^{T}\Omega x\;. \end{array}$$

While pre‐computing Ω according to Equation (6) would factor in the penalty factors for each voxel into the pre‐computation, real planning penalties are set per objective function assigned to volumes‐of‐interest (VOIs). We can align with this practice by separating the VOI‐wise summands of Ω without including the penalties:

(7) $$\Omega =\sum_{v}p_{v}\Omega_{v}=\sum_{v}p_{v}\left(E\left[D_{v}^{T}D_{v}\right]-E{\left[D_{v}\right]}^{T}E\left[D_{v}\right]\right)$$

Here, v indicates a volume of interest, and $\Omega_{v}$ can now be used as the basis of any number of robustness objectives defined on VOI v.

The heavy matrix products are then carried out, including only the voxels within the respective structure. Once normalized by the structure volume, the quadratic form $x^{T}\Omega x$ represents a *total variance* term associated with the structure and quantifies the uncertainty of the dose distribution. Ω is a square, positive semi‐definite matrix. The largest benefit of introducing Ω is that its size is independent of the number of voxels for optimization, while the most substantial drawback is the loss of voxel‐specific information about the variance.

Now, Equation (4) is, of course, only strictly valid when the squared deviation cost function (Equation 3) is applied. However, such a formulation allows for the interpretation of the two terms appearing in Equation (4) as two independent cost‐functions. The first one encodes a variance‐reduction objective, the second one encodes the dosimetric objective. Following such interpretation, a new optimization problem can be defined by extending the application of such cost‐functions to any dosimetric objective. The scenario‐free optimization approach consists thus in applying the desired cost‐function to the expectation value of the dose distribution (estimated via Equation 5) and in adding independently the variance‐reduction term.

#### 2.2.2 Precalculation of the probabilistic quantities

Note that the term “scenario‐free” only applies to optimization, while during dose calculation, explicit calculation of each error scenario still occurs. However, accumulation of the probabilistic quantities Ω and E[D] can be carried out to avoid retention of individual scenario information in memory. This way, the pure optimization time still remains independent of the number of considered scenarios.

A fundamental role in the definition of the probabilistic quantities is played by the procedure applied to sample the probability distribution modeling the uncertainty source. This work explores both a worst‐case and a random sampling approach. For the first case, 26 shift error scenarios were sampled. 2 additional over‐ and under‐shoot range errors and the nominal scenario were added for a total amount of 29 scenarios included.^11^ For the random sampling approach instead, the number of scenarios can be defined by the user, and sample size analysis was performed to showcase the impact of such choice on plan robustness.

In case a 4DCT dataset is included, two different approaches can be followed for handling the different CT‐phases. Error scenarios can indeed be accumulated either within each CT‐phase or considering the whole pool of error and CT scenarios combined. Both methods were implemented and compared.

### 2.3 Implementation and evaluation

The algorithm was developed and tested within *matRad*.^25^, ^26^ The implemented scenario‐free approach was benchmarked against the traditional expected value optimization method described by Equation (2), including multiple 3D and 4D robust optimization setups. In the following, such algorithm will be referred to as *stochastic*. For the 4D optimization strategies, an additional non‐robust, margin‐based plan was also developed. In this case, to partially preserve target coverage in presence of uncertainty, a suitable margin was applied to the internal target volume (ITV) to obtain the planning target volume (PTV).

Setup error scenarios were obtained by a rigid shift of the isocenter position in all three spatial directions. The translation amplitudes were sampled from a normal probability distribution with a set value of 2.25mm standard deviation.

Range errors were modeled as absolute and relative deviation of the water‐equivalent voxel depths. Specifically, the relative $c_{rel}$ and absolute $c_{abs}$ deviations are applied to the radiological depth $rd_{ij}$ of voxel i for beamlet j following the relation: $rd_{ij}=\bar{rd_{ij}}\cdot c_{rel}+c_{abs}$, where $\bar{rd_{ij}}$ is the nominal radiological depth. Such approach is default in matRad for modeling scenarios and is derived from the quantification of range uncertainty as an absolute and relative component for pencil‐beam algorithms by Paganetti.^27^ Ultimately, the choice of relative or absolute components is up to the planner. Range scenarios were then sampled from normal probability distributions with standard deviations of 1mm and 3.5% for the absolute and relative components, respectively.

For the worst case sampling, the same probability distributions were assumed with the scenarios collected at ±2σ both for setup and range errors. For the scope of this analysis, all CT‐phase scenarios included for the 4D optimization were obtained at equidistant time points and can thus be assumed as “equally likely,” translating to a uniform scenario distribution and thus uniform weights as well.

Because of the finite size of the scenario sample set, the expectation value operators introduced in Section 2.1 are approximated with the weighted average from Equation (2). The normalized importance weights $\pi_{s}$ are determined by the specific sampling technique used to generate the scenario set. In case of the worst‐case sampling technique, the importance weights are computed as the probability of the scenario s according to the aforementioned probability distributions' density function. On the contrary, when random sampling is applied, the relative importance of each error scenario is already represented by the frequency of its appearance in the sample set. Thus, the relative importance weight $\pi_{s}$ in the expected value operator is equal for all scenarios.

For each optimized plan, robustness analysis was performed evaluating the scenario‐specific dose distribution with the optimized set of fluence weights. Robustness was quantified through standard‐deviation‐volume‐histograms (SDVH). An SDVH relates standard deviation (SD) to percentage volume and is used to graphically represent the impact of uncertainty within a given structure. Additionally, dose‐volume‐histogram (DVH) bands, dose, and SD distributions are reported for a subset of cases.

The computational efficiency in solving the optimization problem was estimated in terms of time per iteration (TPI), that is, the average time required to compute one step of the iterative optimization problem. Taking the nominal margin‐based optimization as a reference, performances among different algorithms can be quantified in terms of relative TPI (rTPI).

### 2.4 Treatment plans

Four geometrical models were included to showcase different features of the developed algorithm. These included a simple homogeneous box phantom, both in a 3D and 4D setup, and two lung cancer patient 4DCT datasets. The homogeneous box phantom includes a central box‐shaped target the HU values of which have been increased to simulate a difference in density between target and OARs. The 4D dataset for the box‐shaped phantom was obtained by artificially simulating a horizontal organ motion of the 3D box‐phantom through multiple CT‐phases. A total amount of 10 CT phases were generated in steps of 3mm shifts along a single direction. The CT phases were thus uniformly distributed over a total amplitude of motion of 30mm spanning between ± 15mm. For both the 3D and 4D setups, the resolution for dose calculation, that is the voxel dimension, was set to 3mm.

The patient datasets were selected from the Cancer Imaging Archive.^28^ Geometrical specifications of the investigated phantoms and patient cases are summarized in Table 1. Plans were optimized for both photon and proton irradiation. For the proton cases, a constant RBE factor of 1.1 was applied. The dose calculation resolution was set to 3mm for all the proton and photon cases, with the exception of the first patient case's proton plan. In this case, both a 3 and 2mm resolution were applied to further address the memory storage requirements of the algorithms. The robustness analysis for this plan is reported only for the 2mm grid.

**TABLE 1:** Properties of the different geometrical setup used.

Phantom	#CTs	Field angles (*p* 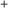 )	Field angles (γ)	ITV‐margin
BOX	1	0 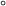 , 90 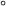	—	—
4D‐BOX	10	0 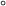 , 90 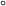	$0^{\circ }$, $51^{\circ },102^{\circ },154^{\circ },205^{\circ },257^{\circ }$, $308^{\circ }$	4mm
Patient 1	10	$45^{\circ }$, $90^{\circ }$, $135^{\circ }$	$0^{\circ }$, $51^{\circ },102^{\circ },154^{\circ },205^{\circ },257^{\circ }$, $308^{\circ }$	4mm
Patient 2	10	$0^{\circ }$, $270^{\circ }$, $315^{\circ }$	$0^{\circ }$, $51^{\circ },102^{\circ },154^{\circ },205^{\circ },257^{\circ }$, $308^{\circ }$	8mm

*Note*: #CTs refers to the number of CT‐phases available, while (*p* 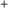) and (γ) refer to proton and photon irradiation, respectively.

Abbreviation: ITV, internal target volume.

The applied cost functions and reference dose prescriptions varied according to the optimization setup implemented. The traditional probabilistic algorithm and the margin‐based approach always apply the cost‐functions to the individual scenario distributions. For all the optimized plans, the scenario‐free approach consistently applies the same dosimetric objectives to the expected dose distribution. Robustness is achieved via additional variance reduction objectives or constraints, the relative importance of which is tuned accordingly. An overview of the optimization configurations used is reported in Table 2. The direct optimization result using these functions is reported and no additional normalization of the optimized dose distribution to a prescribed dose was applied.

**TABLE 2:** Summary of the optimized plans for all phantoms, including the number of scenarios used for optimization, and the optimization algorithm applied: scenario‐free (s‐f), stochastic (s), and margin‐based (m‐b).

**Case**	**Cost‐functions**	**Prescriptions**	**Algorithms**	**Scenarios**	**Phantom**
Algorithm validation	(T) $f_{sqdev}$, $f_{meanVar}$	60Gy, $0\;\;{\mathrm{Gy}}^{2}$	s, s‐f	9	BOX
	(O) $f_{sqdev}$, $f_{meanVar}$	0Gy, $0\;\;{\mathrm{Gy}}^{2}$			
Variance constrained optimization	(T) $f_{sqdev}$	60Gy	s, s‐f	9	
	(O) $f_{sqdev}$, $f_{meanVar}$	0Gy, $0\;{\mathrm{Gy}}^{2}$			
	(T) $c_{meanVar}$	(a) $2.9\times 10^{-2}\;\;{\mathrm{Gy}}^{2}$			
		(b) $2.9\times 10^{-3}\;\;{\mathrm{Gy}}^{2}$			
Worst‐case versus random sampling	(T) $f_{sqdev}$, $f_{meanVar}$	60Gy, $0\;\;{\mathrm{Gy}}^{2}$	s, s‐f	29	
	(O) $f_{sqover}$, $f_{meanVar}$	30Gy, $0\;\;{\mathrm{Gy}}^{2}$			
Sample size	(T) $f_{sqdev}$, $f_{meanVar}$	60Gy, $0\;\;{\mathrm{Gy}}^{2}$	s‐f	5, 29,	
analysis	(O) $f_{sqover}$, $f_{meanVar}$	30Gy, $0\;\;{\mathrm{Gy}}^{2}$		50, 100	
4D Scenario accumulation	(T) $f_{sqdev}$, $f_{meanVar}$	60Gy, $0\;\;{\mathrm{Gy}}^{2}$	s‐f	30	4D‐BOX
	(O) $f_{sqover}$, $f_{meanVar}$	30Gy, $0\;\;{\mathrm{Gy}}^{2}$			
Plan comparison	(T) $f_{sqdev}$, $f_{meanVar}$	60Gy, $0\;\;{\mathrm{Gy}}^{2}$	s, s‐f, m‐b	30	
	(O) $f_{sqover}$, $f_{meanVar}$	30Gy, $0\;\;{\mathrm{Gy}}^{2}$			
Plan comparison	(T) $f_{sqdev}$, $f_{meanVar}$	50Gy, $0\;\;{\mathrm{Gy}}^{2}$	s, s‐f, m‐b	30	P1
	(O) $f_{sqover}$	25Gy			
Plan comparison	(T) $f_{sqdev}$, $f_{meanVar}$	60Gy, $0\;\;{\mathrm{Gy}}^{2}$	s‐f, m‐b	100	P2
	(L) $f_{maxDVH}$	$D_{46}$ = 30Gy			
	(L) $f_{mean}$	0Gy			
	(L) $f_{meanVar}$	$0\;\;{\mathrm{Gy}}^{2}$			
	(L) $c_{maxmean\;dose}$	$20\;\;{\mathrm{Gy}}^{2}$			
	(SC) $f_{mean}$, $f_{meanVar}$	0Gy, $0\;\;{\mathrm{Gy}}^{2}$			
	(B) $f_{sqover}$	60Gy			

*Note*: BOX and 4D‐BOX refer to the box‐shaped phantom in the 3D and 4D configuration respectively, while P1 and P2 refer to the two 4D patient cases. According to the specific phantom, the cost‐functions are applied to different structures referred as: Target (T), OAR (O), Lungs (L), Spinal Cord (SC), and Body contour (B). The applied cost functions are reported as defined in matRad,^25^ with the additional mean variance objective and constraints ($f_{meanVar}$, $c_{meanVar}$) and a constraint set for the maximum value of mean dose ($c_{maxmean\;dose}$). The squared‐deviation and mean‐Variance cost functions have been abbreviated as $f_{sqdev}$ and $f_{meanVar}$, respectively. All the cost functions are normalized by the number of voxels within the considered structure.

## 3 RESULTS

### 3.1 Box phantom

#### 3.1.1 Algorithm validation on the box‐phantom

The first plan was optimized for validation purposes on the box phantom. It uses two proton beams and was optimized both with the scenario‐free and the traditional stochastic robust algorithm. The applied cost‐functions are pure penalized least‐squares objectives of the form expressed by Equation (4). Additionally, a nominal non‐robust plan was also optimized for comparison using Equation (3). In order to enhance the impact of uncertainty, no additional planning margin was added for this plan to the target structure to obtain the PTV.

Robustness analysis was performed on the optimized plans over a pool of 100 setup and range error scenarios randomly sampled from the same probability distribution used to accumulate the probabilistic quantities.

**FIGURE 1:**
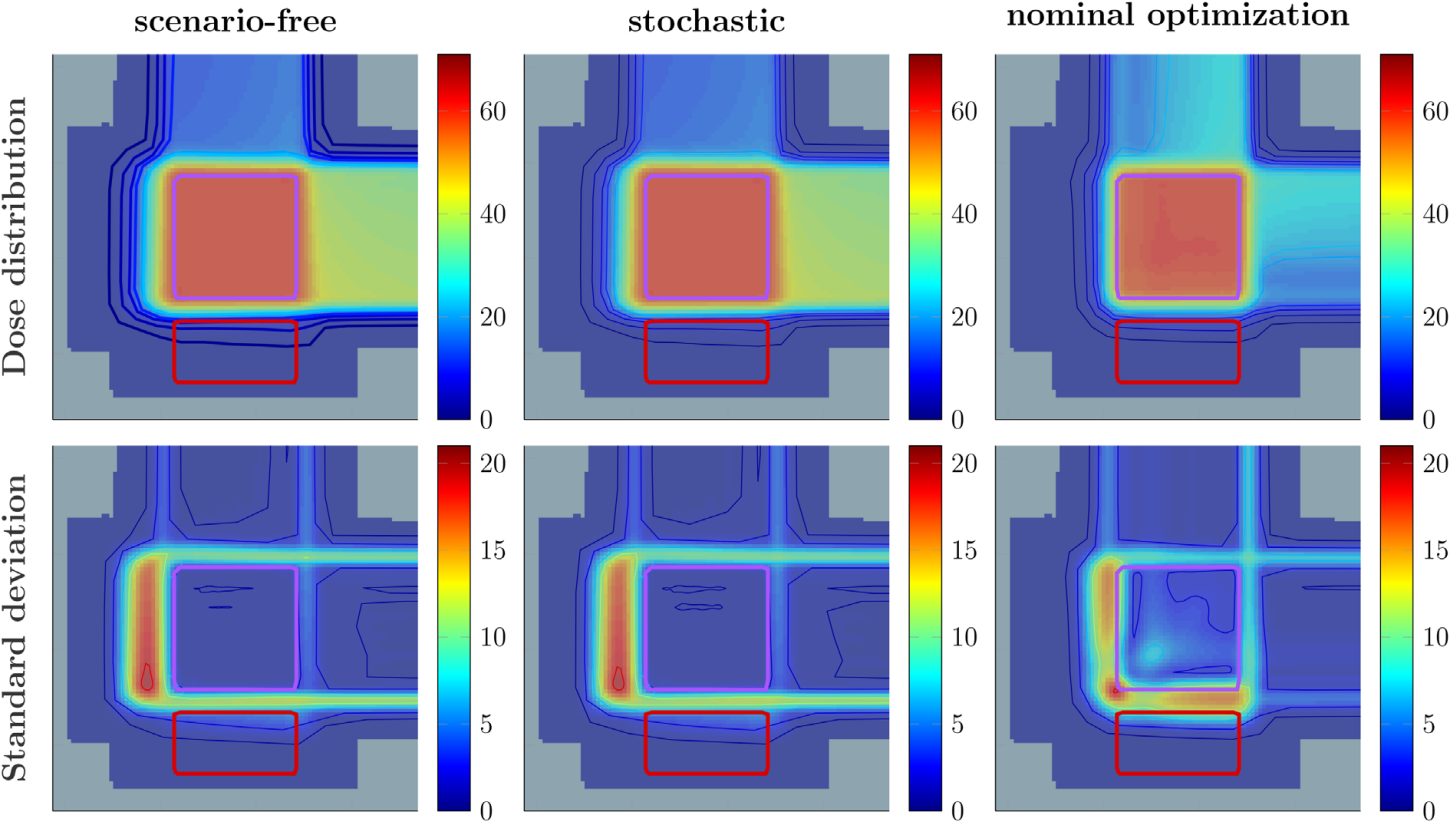
Expected dose (top) and corresponding SD (bottom) obtained with the scenario‐free (left), traditional stochastic (middle) and nominal (right) optimization approaches for the 2 fields proton plan. The target structure and the OAR are contoured in purple and red, respectively. All the colorbar values are reported in Gy.

The obtained dose and standard deviation distribution for the three algorithms are depicted in Figure 1 for a single isocentric slice of the phantom.

Both the robust optimization algorithms achieved a low and uniform distribution of uncertainty within the target structure. In this case of pure penalized least‐squares objective functions, both optimization problems (i.e., the objective functions) are symbolically equivalent, yet differ in their numerical evaluation. Still, a global γ‐analysis (1mm/1% at 1% expected‐dose and SD threshold) confirmed similar results with a 100% pass‐rate for both dose and SD distributions. The nominal reference plan, as expected, showed higher values of SD within the target and toward the distal range for both fields.

**FIGURE 2:**
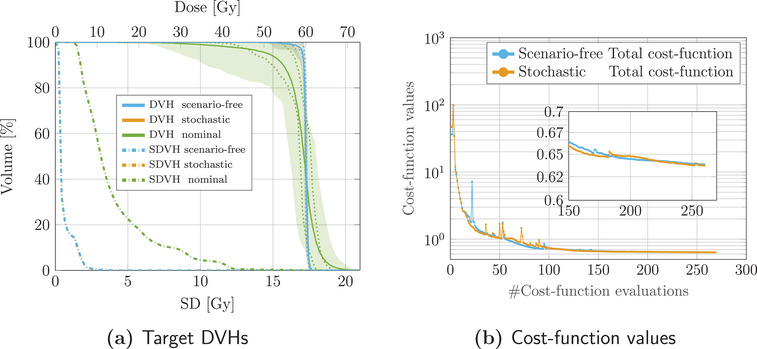
Dosimetric quality and cost‐function evolution for the validation of the scenario‐free algorithm compared to the conventional stochastic implementation. (a) DVHs and SDVHs for the target structure and all applied algorithms. The solid line represents the DVH computed for the expected dose distribution, while the dotted lines correspond to the 25‐75 percentiles of single‐scenario DVHs distributions.The reported DVH bands extend to the 5‐95 percentiles. The uncertainty bands for the two robust approaches are here much reduced with respect to the nominal optimization uncertainty band and overlap in a narrow area around the DVH solid line. The blue and orange dotted lines for the SDVH are also overlapping and indistinguishable due to the equivalence of the two robustness approaches in this specific optimization configuration. (b) Values of the cost‐functions for each performed iteration for the two robust algorithms. DVH, dose‐volume‐histogram; SDVH, standard‐deviation‐volume histograms.

Figure 2 reports the DVHs and SDVHs analysis performed on the target structure for both robust optimization strategies and the nominal plan. This confirms the high target dose uncertainty observed in the nominal reference plan, while the equivalent stochastic and scenario‐free approaches show reduced, nearly equal expected DVH and confidence bands. Particularly, in Figure 2 results for the two robust optimization strategies strongly overlap both in the DVH distributions and in the SDVH lines. The difficulties in distinguishing the two results further confirm the equivalency of the approaches. The evolution of the objective functions through the iterations of the algorithm is depicted in Figure 2, further highlighting the equivalence of the robust optimization algorithms.

Although the absolute optimization time strongly depends on the specific implementation and available hardware, the rTPI for the two robust plans can still be compared. For this plan, the rTPIs for the scenario‐free and stochastic approaches were 1.7 and 8.3, respectively.

The memory footprint of this implementation is largely defined by resolution and corresponding number of voxels and beamlets, as well as the sparsity structure of the dose influence matrices/Ω matrices.

For the phantom with approximately $1.4\times 10^{7}$ voxels, the 2‐field proton plan included approximately $1.9\times 10^{4}$ beamlets. The influence matrices were stored in MATLABs compressed‐column (CSC) format, requiring storage of $n_{nz}$ 64‐bit integer indices, $n_{nz}$ 64‐bit double precision values, and $n_{b}+1$ 64‐bit integer indices to map the non‐zero indices to columns, where $n_{nz}$ is the number of non‐zero values ($2.5\times 10^{8}$ for this case) and $n_{b}$ is the number of beamlets (i.e., matrix columns). Given this storage scheme and numbers, a *single* matrix (in this case the expected dose influence matrix) is required 3.8 GB of storage. For comparison, the combined memory usage for all Ω matrices was 4.1 GB (double precision).

The average time required to perform a single dose‐influence matrix product was approximately 165ms. The time required for a single total variance product was instead of 58ms.

In the following, the memory usage for the E[D] and Ω is reported for each relevant case.

#### 3.1.2 Constrained scenario‐free optimization

**FIGURE 3:**
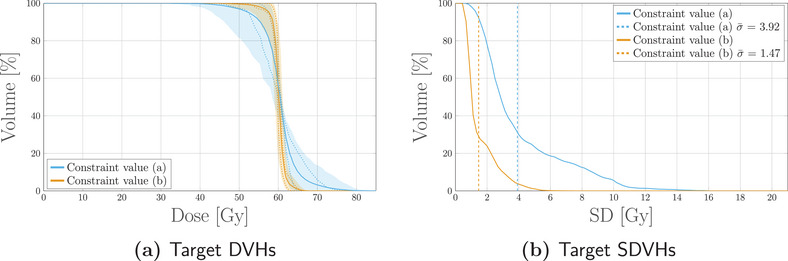
DVH (a) and SDVH (b) comparison for the target structure when two different constraint values are applied to the “mean variance” term in the cost‐function. The two constraints correspond to the values of ${\bar{\sigma }}_{a}^{2}=2.9\times 10^{-2}\;\;{\mathrm{Gy}}^{2}$ and ${\bar{\sigma }}_{b}^{2}=2.9\times 10^{-3}\;\;{\mathrm{Gy}}^{2}$ referred to as Constraint value (a) and Constraint value (b) in the figure legend. The solid line in (a) represents the DVH computed for the expected dose distribution, while the dotted lines correspond to the 25–75 percentiles. The colored band spans instead the 5–95 percentiles. The dotted vertical lines in (b) represent the mean standard deviation, the value of which is also reported in the figure legend for clarity. DVH, dose‐volume‐histogram; SDVH, standard‐deviation‐volume histograms.

The second experiment on the box phantom validates constrained variance minimization. Two plans were optimized, setting mean variance constraints for the target structure with upper thresholds of ${\bar{\sigma }}_{a}^{2}=2.9\times 10^{-2}\;\;{\mathrm{Gy}}^{2}$ and ${\bar{\sigma }}_{b}^{2}=2.9\times 10^{-3}\;\;{\mathrm{Gy}}^{2}$, respectively. Results for this configuration are reported in Figure 3.

As expected, posing a tight constraint on the mean variance results in a shift toward lower values of average standard deviation. At the same time, the distribution of DVHs also cluster around the dose prescription of 60Gy.

#### 3.1.3 Algorithm extension and sampling technique analysis

For the next experiment, cost‐function definitions beyond the pure least‐square objective are applied to transition to more representative planning scenarios. This violates the formal equivalency expressed by Equation (4) such that scenario‐free optimization is no longer equivalent to the scenario‐based stochastic approach. Further, the impact of choosing other common sampling techniques using gridded scenarios (i.e., predetermined shifts over random samples) on the scenario‐free approach is illustrated.

Two plans were optimized with the scenario‐free approach exploiting a random and worst‐case sampling technique. The worst‐case scenarios were selected as described in Section 2.2.2, including a total of 29 error scenarios. For compatibility, the sample size for the random sampling was also set to 29 scenarios. The probabilistic quantities were then computed on both sets. For additional comparison, a third plan was optimized with the stochastic approach using the same set of randomly sampled 29 error scenarios.

Subsequently, to asses the impact of the sample size, three additional sets of probabilistic quantities were computed with a sample size of 5, 50, and 100 randomly sampled scenarios. For this sample size analysis, plans were optimized for the scenario‐free algorithm only.

For all the optimized plans, robustness analysis was subsequently performed on a pool of 100 randomly sampled error scenarios.

**FIGURE 4:**
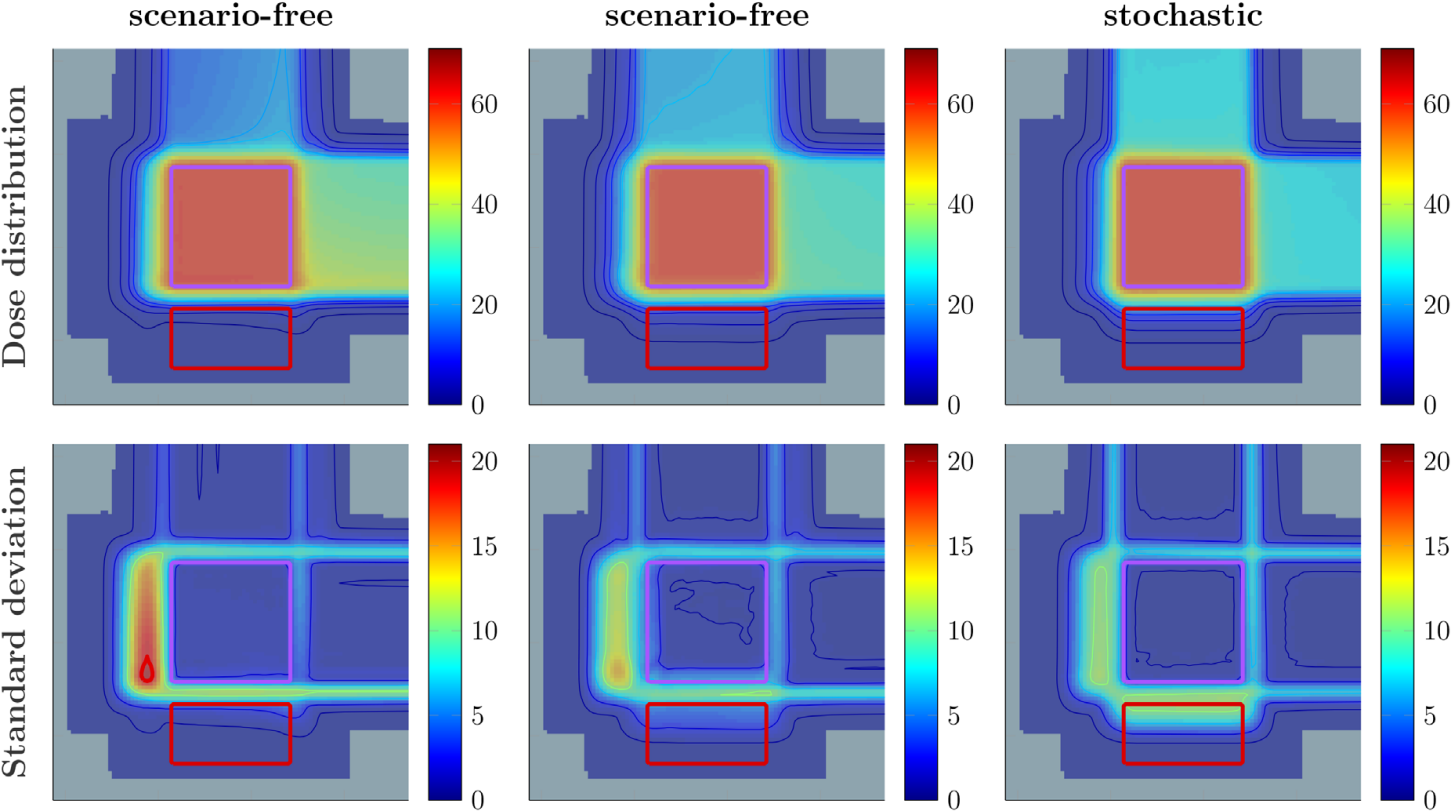
Expected dose and SD distributions obtained with the different approaches and scenario sampling techniques described in Section 2.2.2. Top row: expected dose distributions obtained with the scenario‐free algorithm and a randomly sampled set of scenarios (left), the scenario‐free algorithm and a worst‐case sampling approach (middle), the stochastic algorithm applied to the same set of randomly sampled scenarios (right). Bottom row: the corresponding standard deviation distributions. All colorbar values are reported in Gy. The purple and red contours reported in all figures delineate the box‐shaped Target structure and OAR, respectively.

Figure 4 reports the expected dose and SD distributions obtained with the different sampling techniques and optimization algorithms. As expected, the scenario‐free and stochastic results are no longer matching, since the scenario‐free approach uses direct variance minimization independent of the (expected) dose objectives used on the VOIs. Both the plans obtained with the scenario‐free algorithm showed a lower SD distribution in the OAR compared to the stochastic optimization. This latter exhibits more balanced contribution of the two beams, as highlighted by the dose values in the entrance channels and the symmetric distribution of uncertainty.

The random‐sampling plan applied to the scenario‐free approach showed a better sparing of the OAR from uncertainty when compared to the worst‐case sampling approach, and resulted in a higher distribution of dose and SD delivered by the 90 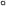 proton field.

**FIGURE 5:**
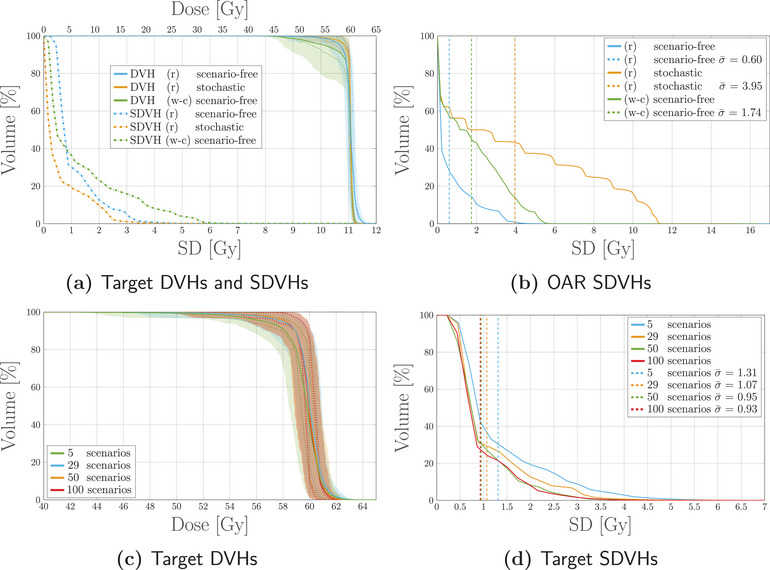
Robustness analysis for the different sampling and optimization approaches. DVHs and SDVHs (a) for the target structure and SDVHs (b) for the OAR reported for both the sampling procedures applied to the scenario‐free approach and the additional stochastic approach. The two sampling procedures are referred to in the legend as (r) for the random sampling and (w‐c) for the worst case sampling. Target DVHs (c) and Target SDVHs (d) for the scenario‐free optimization when different sample sizes are used. The 50 and 100 scenario cases have a considerable overlap both in (c) and (d). For the reported DVHs in (a) and (c), the solid line represents the DVH computed for the expected dose distribution, while the dashed lines correspond to the 25–75 percentiles of single‐scenario DVHs distributions. The colored band spans instead the 5–95 percentiles. The dotted vertical lines in the SDVH plot represent the average value of the SD distribution. DVH, dose‐volume‐histogram; SDVH, standard‐deviation‐volume histograms.

Figure 5 displays DVHs and SDVHs for the optimized plans. The average SD values in the target structure resulted in close values of 1.1 and 1.3Gy for the random and worst‐case sampling applied to the scenario‐free approach, respectively. The average SD value for the stochastic plan was 0.6Gy. The shape of the SDVH, however, shows how the worst‐case sampling allows for a limited amount of voxels to reach high SD values. The SDVH for the OAR is shifted to larger values for the scenario‐free worst‐case sampling, and the stochastic algorithm in order, as already highlighted by the distributions in Figure 4.

The robustness analysis in Figure 5 collects the DVHs and SDVHs obtained with the scenario‐free algorithm for different sizes of scenarios sample. While no considerable difference in the distribution of scenario DVHs can be observed, the SDVHs present a clear trend of mean standard deviation reduction with the increase of sample size.

Probabilistic quantities accumulated for 5, 50, and 100 scenarios resulted in similar memory requirements. In particular, the storage required for the expected dose influence matrix was 3.4, 4.7, and 5.0 GB, respectively. The increasing storage requirement with the number of scenarios can be understood considering the accumulation process over the error scenarios. The different single‐scenario dose distributions will necessarily involve voxels at different locations and indexes and each non‐zero value in any scenario will translate in a non‐zero value in the expected dose influence matrix. Additionally, the total storage needed for the Ω matrices was of 3.7, 4.7, and 4.8 GB for the three cases, respectively.

### 3.2 4D box

As the scenario‐free approach is expected to show its biggest benefit in high‐dimensional uncertainty models, application to 4D planning was evaluated. Artificial target motion was initially simulated via multiple CT‐phases of the previous box geometry. The complete set of scenarios includes thus both error scenarios and CT‐phases. Unless explicitly specified, for all 4D setups the target structure for robust optimization coincides with the CTV of each CT‐phase. The target for the nominal optimization is instead always a PTV obtained by margin expansion of the ITV as indicated in Table 1. During robustness analysis instead, each distribution, DVH and SDVH is evaluated on the scenario‐specific structures, that is the CTV is used as a target structure.

**FIGURE 6:**
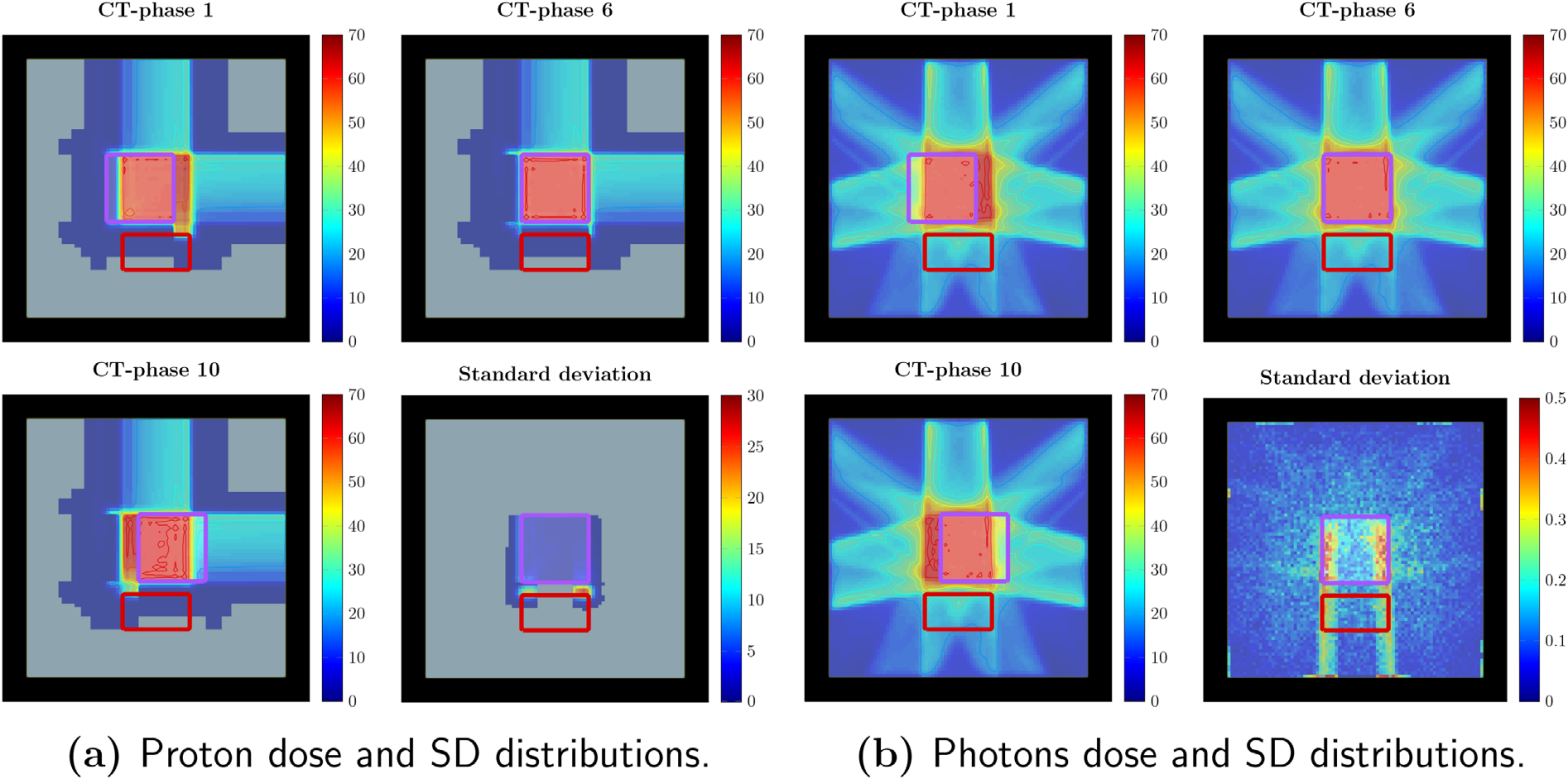
Dose distributions obtained for the CT‐phases at the lower (CT‐phase 1) and upper (CT‐phase 10) end of the motion range, nominal non‐deviated phase (CT‐phase 6) and the relative standard deviation distribution. The plans correspond to the proton (a) and photon (b) irradiation plans. The dose distributions are obtained as part of post‐optimization robustness analysis performed on the nominally optimized distribution. The contoured structures correspond to the Target structure (purple) and the distal organ at risk (red). All colorbar values are reported in Gy.

In addition to the previous analysis, IMRT plans were also generated and allowed for the assessment of the algorithm performances for photon irradiation as well. Figure 6 illustrates phase dose distributions and their standard deviation for a nominal optimized plan recalculated on the CT phases.

Three plans were optimized for each radiation modality applying the scenario‐free, the traditional stochastic, and a nominal, margin‐based algorithm. Least‐square and squared‐overdosing objectives were applied consistently to the target structure and OARs.

**FIGURE 7:**
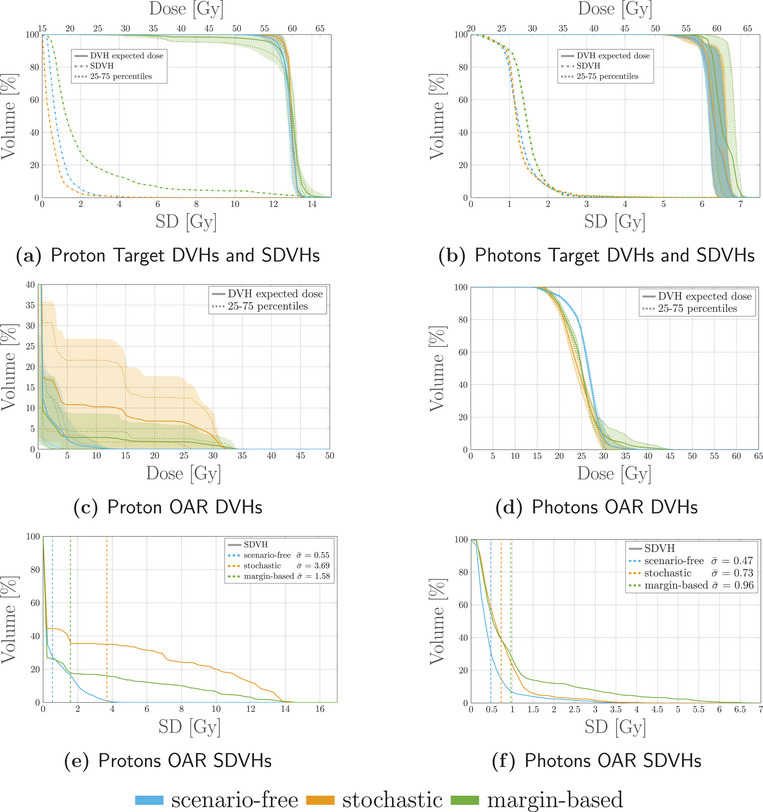
Dosimetric and robustness analysis performed for the photon and proton plans optimized with the scenarios‐free (blue), traditional stochastic (orange) and margin‐based (green) methods for the 4D box‐shaped phantom. Top: DVH and SDVH for the target structure for the proton (a) and photons (b) irradiation plans. Middle: DVHs for the OAR for proton (c) and photons (d) irradiaiton. Bottom: SDVHs for the OAR for proton (e) and photons (f) irradiaiton. For the reported DVHs, the solid line represents the DVH computed for the expected dose distribution while the dotted lines correspond to the 25–75 percentiles of single‐scenario DVHs distributions. The colored band spans instead the 5–95 percentiles. The SDVHs in (a) and (b) are instead reported in dash‐dotted lines. The dotted vertical lines in the SDVH plot represent the mean standard deviation. DVH, dose‐volume‐histogram; SDVH, standard‐deviation‐volume histograms.

Robustness analysis was performed on the optimized plans and comparison between DVHs and SDVHs is reported in Figure 7. As a general remark, the obtained proton plan tends to achieve similar target coverage to the photon plan. The observed dose delivered to the OAR was consistently lower for all three optimization methods for the proton plan, as opposed to the photon irradiation, while the opposite is true for the SDVH curves. Even though comparison between radiation modalities goes beyond the scope of this analysis, the expected behavior is observed, with the proton dose distribution being more conformal to the target and, concurrently, more sensitive to uncertainty than the photon distribution.

For both modalities the robustness in target coverage was higher for both the robust algorithms when compared to the margin‐based optimization. This feature is highlighted by the SDVH curves depicted in Figure 7. A more robust target coverage could be achieved with the nominal optimization by increasing the ITV margin, at the cost of increasing the integral dose and with the risk of delivering higher doses to the surrounding OARs as well.

For the proton plan, the scenario‐free approach was capable of achieving lower dose and SD within the OAR when compared to the traditional stochastic algorithm, while maintaining sufficient target coverage and robustness. This may be attributed to the explicit variance reduction objectives introduced by the scenario‐free algorithm; since the applied cost‐functions are not limited to pure least‐square, higher penalization of the variance is expected.

**FIGURE 8:**
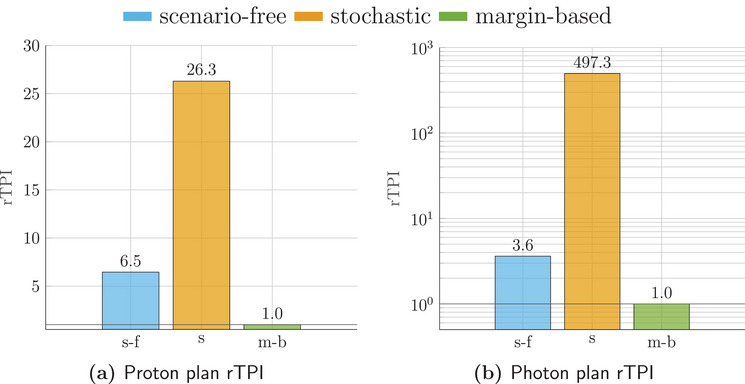
Relative time per iteration (rTPI) for the proton (left) and photons (right) 4D robust plans. The abscissa labels refer to the scenario‐free (s‐f), stochastic (s) and margin‐based (m‐b) approaches. The photon rTPI is reported in logarithmic scale for clarity.

The runtime efficiency for the three algorithms was assessed in terms of rTPI. Considering the TPI for the margin‐based plan as a reference value, rTPI for the scenario‐free and traditional stochastic optimization algorithms are reported in Figure 8 for both radiation modalities.

For both radiation modalities, the observed rTPI for the scenario‐free algorithm was considerably lower than the stochastic approach.

The observed memory storage required for the expected dose influence matrix in the proton and photon cases was of 1.1 and 3.7 GB, respectively. The total storage required for the Ω matrices was instead of 5.2 GB and 307 MB. Despite the higher number of beamlets ($2.3\times 10^{4}$ for the proton case and $2.7\times 10^{3}$ for the photon one), the lower storage required for the expected dose influence matrix of the proton plan has to be attributed to the characteristic single beamlet dose distribution and ultimately, to the finite range of the proton depth dose kernel. The dimensionality of the Ω follows instead an opposite trend and is dominated in this case by the number of beamlets included and their correlation.

**FIGURE 9:**
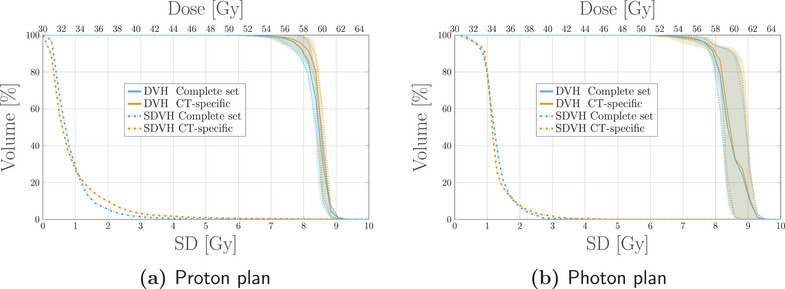
Dosimetric and robustness analysis performed for the two calculation strategies described in Section 2.2.2 for the 4D scenario accumulation. DVHs and SDVHs are obtained for the target structure with the two calculation approaches for proton (a) and photon (b) irradiation. For the reported DVHs, the solid line represents the DVH computed for the expected dose distribution while the dotted lines correspond to the 25–75 percentiles of single‐scenario DVHs distributions. The colored bands span the 5–95 percentiles. DVH, dose‐volume‐histogram; SDVH, standard‐deviation‐volume histograms.

Figure 9 reports a comparison between plans optimized with the scenario‐free approach. The two plans correspond to the different modes described in Section 2.2.2 to compute and combine the probabilistic quantities on multiple CT‐phases. Both plans achieve similar target coverage and robustness.

### 3.3 4D patient case

The characterization of the scenario‐free algorithm performances was also extended to more clinically realistic patient plans. For this analysis a 4D lung cancer patient dataset comprising of 30 scenarios in total was selected. This included 10 CT‐phases modeling the breathing motion and 3 error scenarios for each phase as described in Section 2.4. Even in this case both protons and photons were applied as radiation modalities.

**FIGURE 10:**
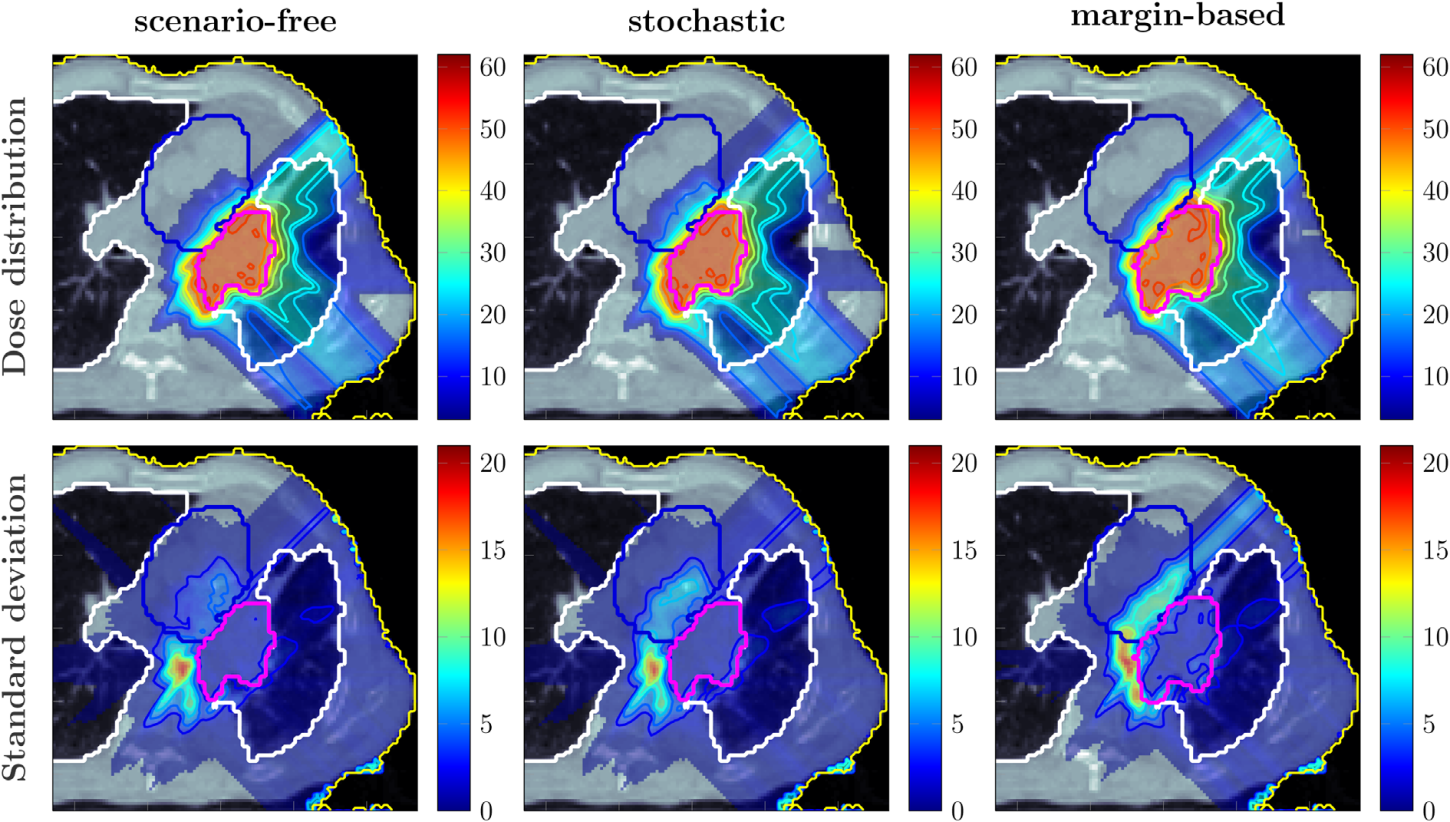
Comparison between proton dose distributions (top row) and SD distributions (bottom row), both reported in Gy, obtained with the scenario‐free (left), the stochastic (middle) and margin‐based (right) optimization algorithms. The contoured structures correspond to the lungs (white), the heart (blue), and the target structure (purple). The target structure coincides with the CTV for the robust optimization algorithms, and with the PTV for the margin‐based plan. CTV, clinical target volume; PTV, planning target volume.

**FIGURE 11:**
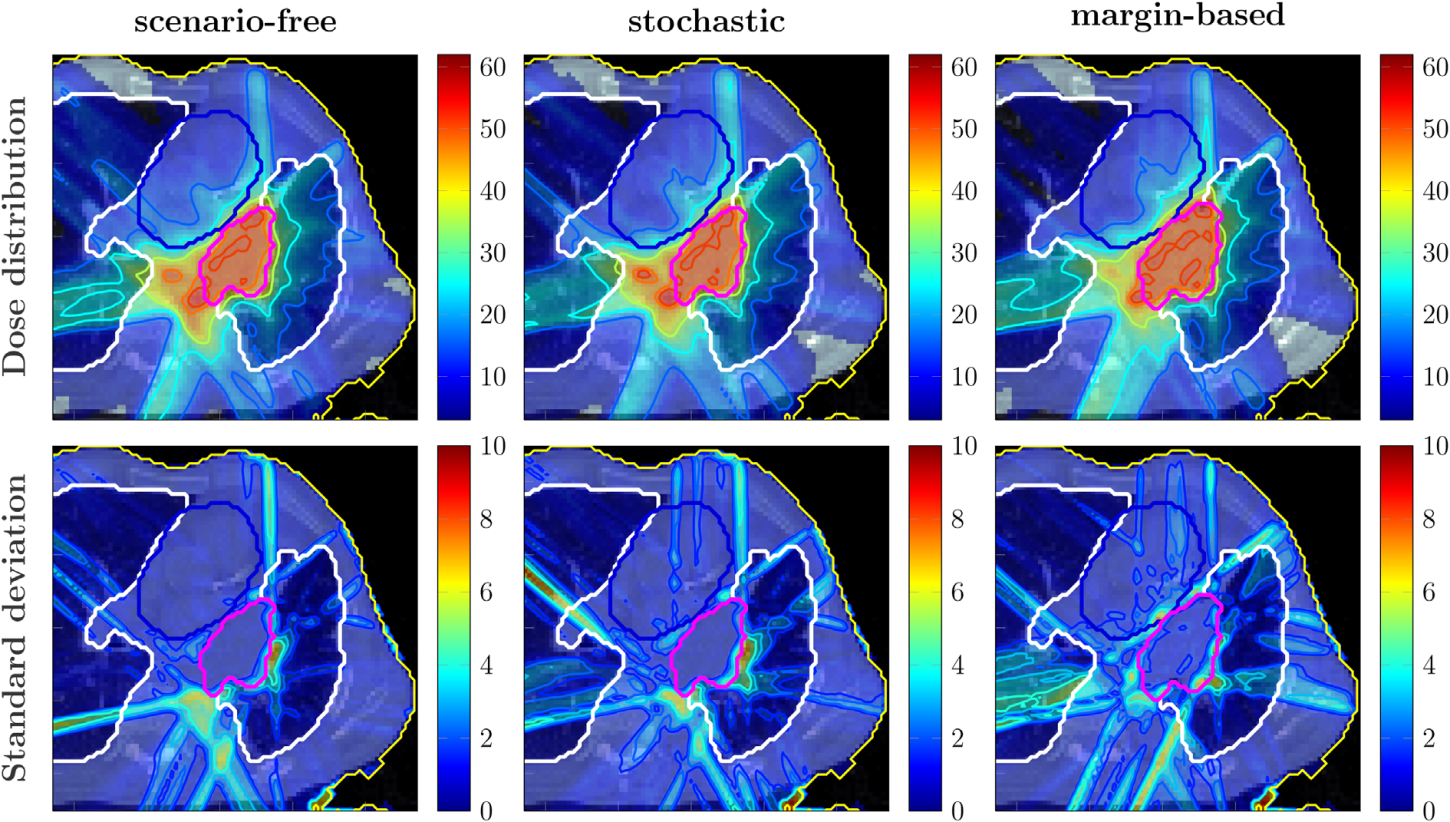
Comparison between photon dose distributions (top row) and standard deviation distributions (bottom row), both reported in Gy, obtained with the scenario‐free (left), the stochastic robust (middle) and margin‐based (right) optimization algorithms. The contoured structures correspond to the lungs (white), the heart (blue), and the target structure (purple). The target structure coincides with the CTV for the robust optimization algorithms, and with the PTV for the margin‐based plan. CTV, clinical target volume; PTV, planning target volume.

Figures 10 and 11 collect examples of dose and standard deviation distributions obtained for the 3‐fields proton and the 7‐fields photons plans, respectively, applying all three optimization approaches. The margin‐based plan shows the highest SD within the target and the major OARs. On the contrary, the lowest values were observed for the scenario‐free approach.

**FIGURE 12:**
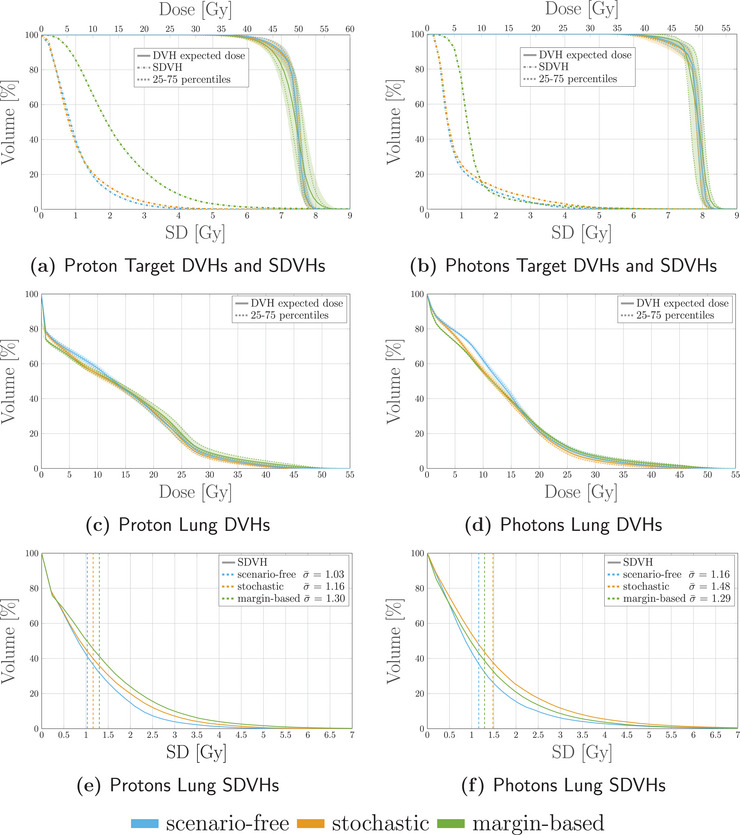
Dosimetric and robustness analysis performed for the photon and proton plans optimized with the scenarios‐free (blue), traditional stochastic (orange) and margin‐based (green) methods. Top: DVH and SDVH for the target structure for the proton (a) and photons (b) irradiation plans. Middle: DVHs for the lung for proton (c) and photons (d) irradiaiton. Bottom: SDVHs for the lung for proton (e) and photons (f) irradiaiton. For the reported DVHs, the solid line represents the DVH computed for the expected dose distribution while the dotted lines correspond to the 25‐75 percentiles of single‐scenario DVHs distributions. The SDVHs in (a) and (b) are instead reported in dash‐dotted lines. The dotted vertical lines in (e) and (f) represent the mean standard deviation. DVH, dose‐volume‐histogram; SDVH, standard‐deviation‐volume histograms.

Robustness analysis was performed, including the dose distributions for all the error scenarios and optimization approaches. Results are collected and represented through DVHs and SDVHs in Figure 12. In this case, the left lung was reported as a representative OAR.

The DVHs for the target structure reported in Figure 12 for the proton plan show an improved and more robust target coverage for the scenario‐free approach. The distribution of single scenario DVHs clusters around the dose prescription with a narrow distribution for both robust algorithms. This is also reflected by the shift of the SDVH curve toward lower values of standard deviation.

Figure 12 reports the DVH and SDVH lines for the same plan and the lung. In this case, the scenario free‐algorithm seemingly tends to slightly enhance the sparing of the organ from high doses and simultaneously reduce the standard deviation. The mean lung dose observed for the scenario‐free, stochastic and margin‐based plans was 13.3±0.5, 16.6±0.5, and 13.20±0.28 Gy, respectively, resulting thus in close values for the scenario‐free and margin‐based approaches, while slightly higher for the stochastic plan.

**FIGURE 13:**
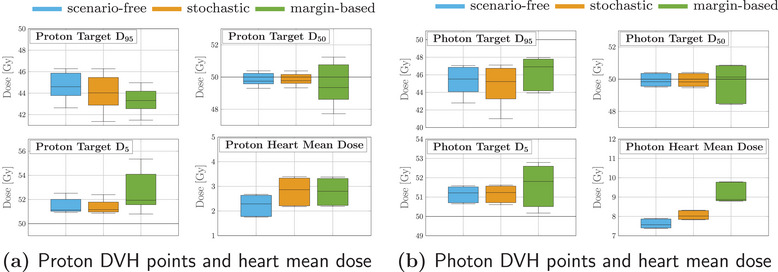
DVH points collected for the proton (a) and photon plan (b). The metrics' values are distributed over the set of error scenarios and reported for all three applied optimization methods. The solid line at 50Gy represents the dose prescription. Reported are also the heart mean dose for both modalities. DVH, dose‐volume‐histogram.

The same analysis was performed for the photon plan and is also reported in Figure 12. Similar target coverage was achieved by the robust optimization algorithms while the distribution of standard deviation within the target is reduced with respect to the margin‐based approach. For this plan the DVH distributions for the lung showed less variability over the three methods. Figure 13 collects selected DVH points for the target structure and the mean dose values for the heart. The scenario‐free and the traditional stochastic algorithm can achieve better target conformity. Compared to the nominal margin‐based optimization, the box plots for the $D_{95}$, $D_{50}$ and $D_{5}$ values for these two algorithms cluster closer to the dose prescription value of 50Gy. The quartile boundaries are also closer to the median value, reflecting the behavior displayed by the SDVH curves in Figure 12. The observed mean heart doses are also lower for the scenario‐free approach in both radiation modalities.

**FIGURE 14:**
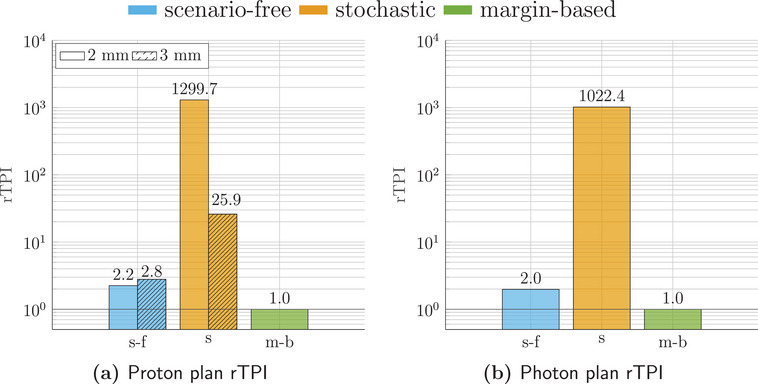
rTPI for the proton (left) and photon (right) lung cancer patient plan. The abscissa labels refer to the scenario‐free (s‐f), stochastic (s) and margin‐based (m‐b) approaches. For the proton case, the results are reported for both 2mm (filled bars) and 3mm (dashed bars) dose grid resolutions. The *y*‐axis is in logarithmic scale. The rTPI values for the 3mm resolution scenario‐free approach result slightly higher than the 2mm case because the reference margin‐base plan was also faster. rTPI, Relative time per iteration.

For this patient, the probabilistic quantities and the error scenarios for the proton plan were computed with a 2mm dose grid resolution. To further analyze the memory footprint and it is dependence on the dose calculation resolution for the three algorithms, Figure 14 adds a comparison with computations on a 3mm dose grid.

For the scenario‐free proton plan, the rTPI increases only slightly, compared to the stochastic approach, when using the worse resolution of 3mm, due to the non‐dependence on the number of voxels in the total variance product. The reduced number of voxels benefits the reference margin plan as well.

For the proton plan, the total number of beamlets used for calculation was of $2.5\times 10^{4}$. The 2mm dose gird resolution case required the storage of 4.6 GB for the expected dose influence matrix, against the 1.0 GB of the 3mm resolution case for the same modality. This resulted in computation times of approximately 200ms and 42ms for the E[D]x product in 2mm and 3mm resolution, respectively, leading to the large rTPI for 2mm resolution. The evaluation time for the quadratic form $x^{T}\Omega x$ (≈82ms) does not change with resolution, the same applying to the storage requirements of 5.5 GB.

For the photon plan, storing all scenario matrices with a 2mm resolution was not possible on our system for the stochastic approach due to the photon plan's transmission beams requiring more storage. With a dose grid resolution of 3mm, the expected dose influence matrix for the photon plan resulted in $3.2\times 10^{3}$ beamlets and 5.2 GB required, already higher than the 2mm resolution proton matrix. Yet the total storage required for the Ω matrices was as low as 412 MB due to the lower number of beamlets.

For the second 4D patient, the number of scenarios was increased to 100, combining 10 range and shift error scenarios for each of the 10 CT phases. Multiple cost‐functions were defined as reported in (Table 2). Only the scenario‐free and the margin‐based approach were applied. Memory limitations in the available hardware prevented the application of the traditional stochastic optimization algorithm with such an high number of scenarios.

**FIGURE 15:**
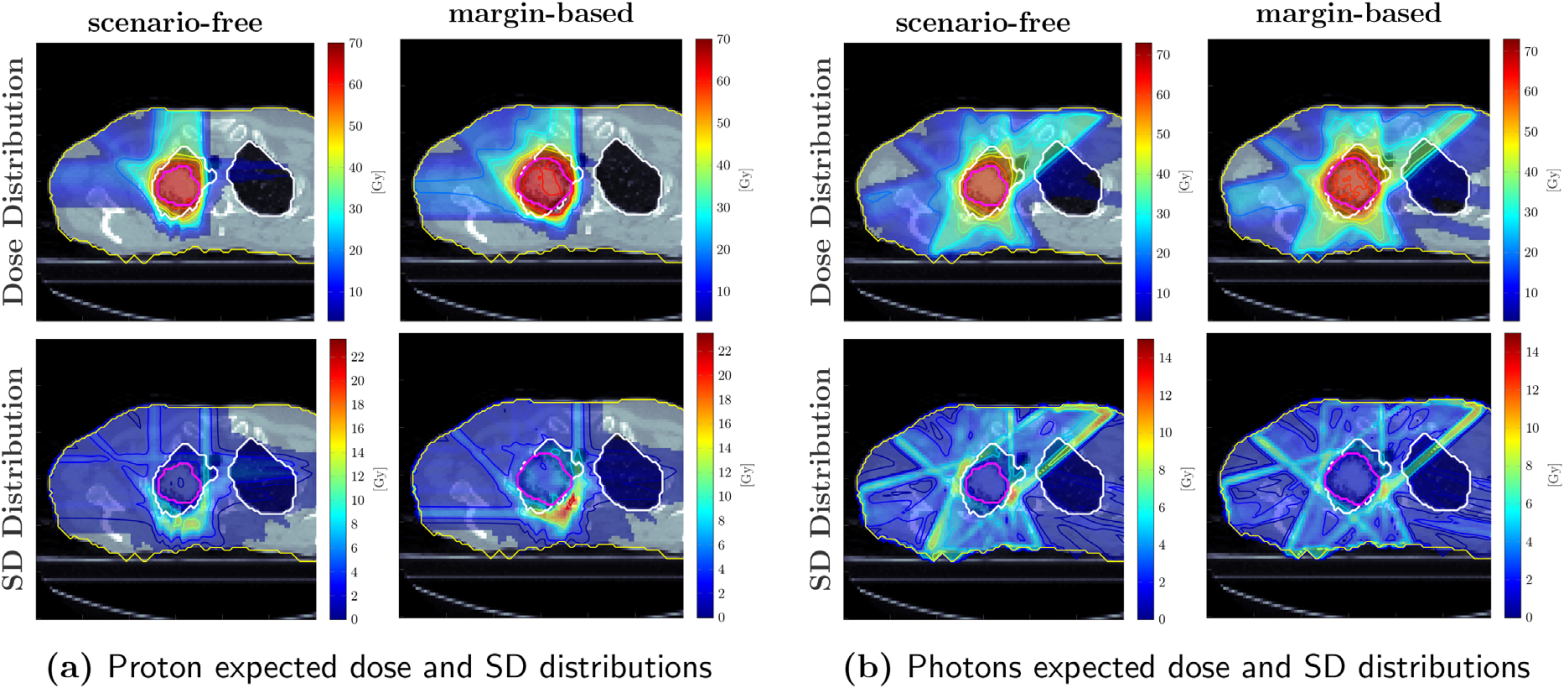
Expected dose (top) and SD (bottom) distributions obtained with the scenario‐free and the margin‐based algorithms for the proton (a) and photon (b) irradiation plan. The contoured structures correspond to the lungs (white) and the target structure (purple). The target structure coincides with the CTV for the robust optimization, and with the PTV for the margin‐based optimization. CTV, clinical target volume; PTV, planning target volume.

**FIGURE 16:**
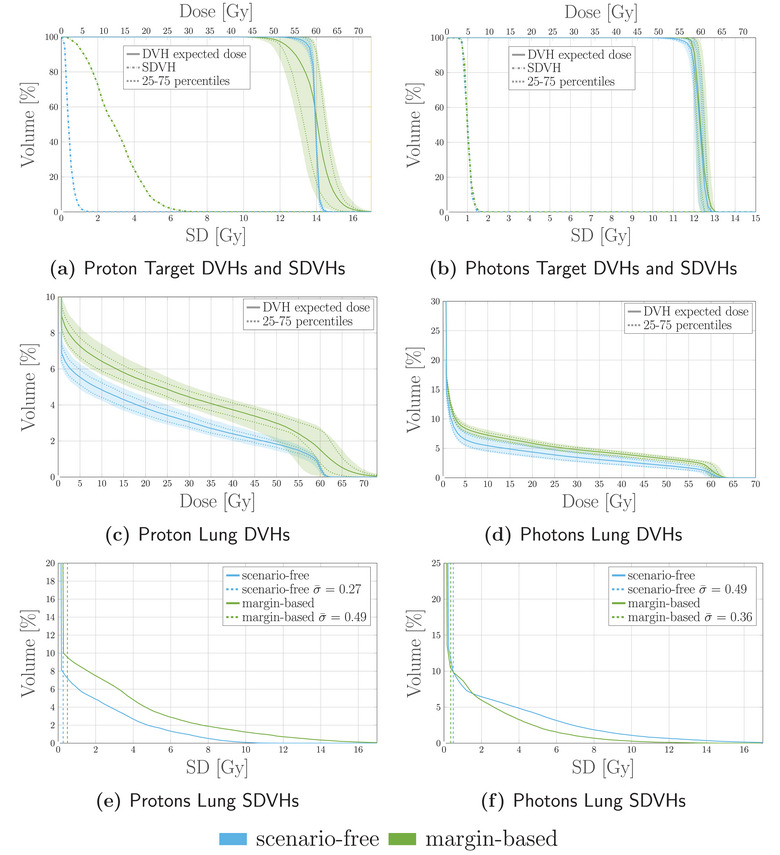
Dosimetric and robustness analysis performed for the photon and proton plans optimized with the scenarios‐free (blue) and margin‐based (green) methods. Top: DVH and SDVH for the target structure for the proton (a) and photons (b) irradiation plans. Middle: DVHs for the lung for proton (c) and photons (d) irradiaiton. Bottom: SDVHs for the lung for proton (e) and photons (f) irradiaiton. For the reported DVHs, the solid line represents the DVH computed for the expected dose distribution while the dotted lines correspond to the 25–75 percentiles of single‐scenario DVHs distributions. The SDVHs in (a) and (b) are instead reported in dash‐dotted lines. The dotted vertical lines in (e) and (f) represent the mean standard deviation. DVH, dose‐volume‐histogram; SDVH, standard‐deviation‐volume histograms.

Figure 15 reports expected dose and SD distributions obtained with the scenario‐free and margin‐based approaches for the proton and photon plans. Additional dosimetric and robustness analysis was performed and reported in Figure 16 for the same plans. For the proton case, the scenario‐free approach was capable of delivering a more conformal dose distribution to the CTV. The SDVH curve for the target structure, as well as the dose delivered to the lung, were also lower with respect to the margin‐based approach.

For the photon case the benefit in terms of target coverage and robustness of applying robust optimization is less evident when compared to the analogous proton plan. Both algorithms are indeed capable of achieving sufficient target coverage and the SDVH curves for the target structure are close to each other. The scenario‐free approach was again capable of reducing the overall dose delivered to the lung, as highlighted by the DVH distribution in Figure 16.

**FIGURE 17:**
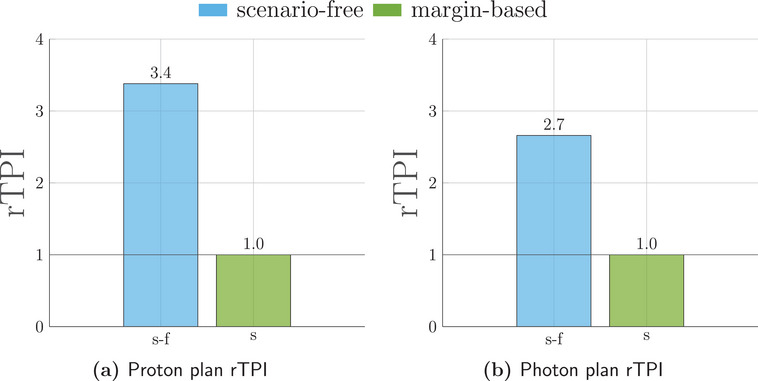
rTPI for the proton (left) and photon (right) lung cancer patient plan. The abscissa labels refer to the scenario‐free (s‐f) and (m‐b) approaches. rTPI, Relative time per iteration.

Figure 17 reports the measured rTPIs for both radiation modalities. Even in this case the scenario‐free approach allows to gain robustness goals with minimal time overhead compared to the nominal optimization.

The memory usage for the proton and photon radiation modalities was of 700 MB and 2.3 GB for the expected dose influence matrix and of 3.3 GB and 158 MB for the Ω matrices respectively. With respect to the previous patient case, the total number of beamlets for both radiation modalities ($1.1\times 10^{4}$ and $1.6\times 10^{3}$, respectively) is lower, as a consequence of the different patient geometry and volume of the tumor site.

## 4 DISCUSSION

The present work demonstrated the applicability of a scenario‐free approach to robust optimization for IMPT and IMRT. The scenario‐free approach uses comparably low‐dimensional, pre‐computed quantities: expected dose influence and total variance influence. During robust plan optimization, scenarios are thus not needed. While, in principle, a fully scenario‐free pipeline can be constructed using, for example, analytical probabilistic modeling,^15^, ^16^, ^19^, ^26^ the scenario‐free approach generalizes to precomputation from conventional error sampling during dose calculation, as used in this work, and is thus compatible with the current state‐of‐the‐art in uncertainty modeling for radiotherapy.

### 4.1 Dosimetric performance

#### 4.1.1 Phantom validation

Validation of the scenario‐free algorithm was conducted exploiting the box‐shaped phantom for both 3D and 4D optimization. Initial comparison between plans optimized with pure least‐square cost‐functions proved the stability of the algorithm behavior. The comparison reported in Figure 1 and Figure 2 highlights the compatibility of the scenario‐free and stochastic algorithms when equivalency of the two approaches is explicit.

The addition of objectives beyond the basic squared‐deviation formulation to the global cost‐function drops the equivalence between the scenario‐free and stochastic approach. This can be understood comparing the distributions and analysis reported in Figures 4 and 5 for the two robustness approaches. Moving from a pure least‐square optimization configuration to non‐zero threshold squared‐overdosing decreases the contribution of the dosimetric objective for the OARs compared to the unchanged variance term, biasing the optimization toward variance reduction.

While this does not have consequences concerning convergence or convexity, it may distort the stochastical meaning due to the separation of expected value and variance. The loss of intuitive interpretation for the stochastic approach as an expected value optimization is compensated by the possibility to directly quantify the uncertainty impact in the form of mean variance.

#### 4.1.2 4D patient cases

After phantom validation, the approach was tested on two realistic lung patients. Scope of this analysis was to benchmark the applicability of the scenario free approach in clinically realistic optimization scenarios.

For the first patient, the analysis reported in Figure 12 highlighted how the scenario‐free approach can achieve similar DVH distributions compared to the traditional stochastic approach, for both radiation modalities. The reported SDVH lines serve as a further confirmation of the effectiveness in reducing the overall uncertainty. Figure 13 highlights a selection of representative DVH metrics, providing additional evidence of the scenario‐free adequateness in realistic setups.

Similarly, the analysis performed for the second patient showcased the feasibility of adopting the scenario‐free algorithm as a full replacement for traditional methods. The robust algorithm achieved both a better and less uncertain target coverage and OAR sparing compared to the corresponding margin based approach. Particularly, the scenario‐free approach succeeded in an optimization setup where the traditional stochastic algorithm could not be applied due to computational limitations.

For both patient cases, the target coverage suggested a less pronounced benefit in applying a robust algorithm, instead of the margin‐based one, when photon irradiation is applied. Comparison between the two modalities is out of the scope of this work however, the reason for this behavior should be found in the static dose cloud approximation, which still holds to some extent for the photon plan when a sufficient PTV margin is considered. On the contrary, similar or reduced mean dose to the OARs was observed for the scenario‐free approach compared to the margin‐based for both patients and radiation modalities.

#### 4.1.3 Uncertainty and robustness analysis considerations

Overall, the scenario‐free approach could compete with the reference methods. Yet, this work did not exhaust the full arsenal of available optimization methods and metrics for uncertainty analysis. Within this work SD maps, DVH bands and SDVHs illustrated the impact on plan robustness of the different algorithms. The effectiveness and reliability of such and other methods is well established in literature.^7^, ^19^, ^29^, ^30^, ^31^, ^32^ Beyond these, robust evaluation metrics as voxel‐wise max‐, min‐ or mean‐dose distributions^6^, ^11^ may help identify hot and cold spots by providing a local information.

Moving toward a more clinically oriented evaluation of the relevant robustness metrics, Korevaar et al.^11^ also highlighted how the correlation between CTV and PTV point metrics can be exploited to define consistent clinical acceptance criteria. Inclusion of such metrics in our work, however, was aggravated through the expansion to 4D robust planning, whose place in clinical operations is yet to be defined. Thus, reporting of SDVHs and DVH bands allowed for a global, more comprehensive quantification of the uncertainty impact in the scope of this work.

Further, the scenario‐free approach allows collapsing multiple sources of uncertainty into one quadratic form. In our approach to 4D optimization, the analysis reported in Figure 9 showcases this possibility by collapsing spatial uncertainties into the 4D phase accumulation. The minimal impact on dosimetric outcome is promising for planning highly complex cases with multiple sources of uncertainty.

While this work only combined spatial uncertainties with anatomical changes and breathing motion, the applicability of the method is however not limited to those. Any source of uncertainty that can be expressed as a sample set of fluence‐related quantity error scenarios should allow for the pre‐calculation of probabilistic quantities and can be approached by the scenario‐free optimization. For example, such matrices could be formed for biological uncertainties as well.^33^

As a general remark, the Ω matrix encodes correlations between beamlets. If the beamlet representation has a direct physical interpretation, as is the case for pencil beam scanning in IMRT, and the scanning pattern is known, the 4D CT‐phases sampling the patient motion can be appropriately coupled with subsets of beamlets to model interplay effects.^34^ By pooling together phase‐specific scenarios for the calculation of the Ω matrices, the impact of interplay effects would also be captured by such construct and minimized.

### 4.2 Computational performance

The computational complexity benefits of the scenario‐free approach (and thus the rTPI) depend on several factors. In general, lower number of beamlets, higher resolutions, and a large number of scenarios favor the scenario‐free approach, as its quadratic forms computational footprint depends quadratically on the number of beamlets only. Especially in 4D robust planning, as shown in this work, the increasing number of scenarios due to the additional time component encourages the use of complexity‐reducing methods like the presented scenario‐free approach.

These aspects were well highlighted by the dimensionality and storage requirements reported for the influence matrices of each case. In particular, the proton plan of the first patient case showed how the optimization time for the scenario‐free approach is independent from the spatial resolution of dose calculation, which only affects the expected‐dose‐influence matrix. Complementary, the second patient showcased how the novel algorithm can be applied regardless of the number of scenarios, which can be as high as 100 without affecting the optimization time. For the second patient, the stochastic approach was instead inapplicable due to the memory limitations of the hardware and implementation.

In general, the observed times reflect the dimensionality of the involved matrices and imply the benefit of trading, with the scenario‐free approach, the computation of a single dose‐influence plus a limited amount of variance‐influence matrix product against several dose‐influence matrices (one for each scenario) required by the traditional approach.

Overall the observed rTPI served as a fair metric to measure and compare the computational efficiency of the optimization methods. In all cases, it resulted in comparable values for the margin‐based and scenario‐free approach. The observed overhead for the scenario‐free originates from the additional calculation of the quadratic form, but always resulted in a favorable trade against the multiple scenario evaluations of the stochastic approach. As a general remark, the application of the scenario‐free approach is only affected by the number of scenarios if scenarios are used for the precomputation of the probabilistic quantities. Although non‐negligible, the additional time required for this accumulation step was always only a fraction of the total time required for dose calculation.

Apart from the general complexity, the specific implementation, like chosen precision and dedicated problem‐specific optimization, as well as the hardware environment (e.g., GPUs or CPUs) will influence the relative performance. Sparse matrix storage and products for the conventional robust planning approach can be dedicatedly optimized for the treatment planning problem,^35^ and in robust planning similar storage patterns between scenarios could be exploited. Yet, such optimizations are very much depending on the matrix structure, and may already be difficult to translate from one radiation modality to the next.

On the other hand, the scenario‐free approach relies on conventional quadratic forms over symmetric positive semi‐definite matrices, all well understood and common mathematical properties, with simple regular storage schemes. This facilitates usage of existing optimized linear algebra codes to reduce calculation time, while a whole arsenal of methods could be used to exploit structure (e.g., symmetric low‐rank matrix decompositions) to further reduce storage.

### 4.3 Outlook

The largest benefit obtained with the scenario‐free approach in optimization time reduction and workflow streamline is therefore achieved when multiple plan optimizations can be performed with the same precalculated probabilistic quantities. This is the case of traditional trial and error penalized weighted‐sum approaches, were the relative importance of optimization objectives and constraints is manually tuned to meet the plan prescriptions.

In the context of this multi‐criteria decision making, the scenario‐free algorithm facilitates decoupling robustness by providing individual objective functions for variance (minimization) and expected dose at minimal computational effort. This separation makes robustness – in the sense of variance – an additional decision criterion in the planning process. The specific objective functions compete with the other conventional objectives and constraints, during optimization, and different trade‐offs between nominal dose and robustness may be explored. Thus, the scenario‐free approach could be used in advanced multi‐criteria optimization approaches like Pareto front approximation and navigation or lexicographic planning. This was already suggested by Chen et al.^36^ using separable variance objectives, but may be substantially improved in performance using the method presented herein.

Addition of specific structure robustifying objectives on top of a nominal optimization could find a potential application in an adaptive planning workflow as well. The daily adapted plan could be obtained with a fast nominal optimization and the voxel‐independent variance reduction chained subsequently. Calculation of the probabilistic quantities could then be based on multiple modeled sources of uncertainties together with the patient specific deviations and inter‐fractional anatomical changes observed through the course of treatment. This would potentially allow the achievement of a stable optimized dose distribution with minimal additional computational effort.

Another potential field of application for the scenario‐free approach is the optimization of Volumetric Modulated Arc Therapy (VMAT) treatment plans. The planning algorithms developed for such technique usually involve substantial computational effort translating to slower optimization times when compared to alternative techniques such as IMRT or Tomotherapy.^37^, ^38^, ^39^ Simultaneously, the improved conformity achievable with VMAT rises concern for the safe delivery of the distribution.^40^ The limited computational demand of the scenario‐free approach could represent a viable solution to perform robust optimization in this context, especially in an initial fluence‐optimization step; as the Ω‐matrices act similar to smoothing matrices, the results might even benefit the VMAT sequencing step. However, the translation to the aperture optimization step might not be straightforward – yet, there is no fundamental roadblock preventing computation of Ω matrices when optimization variables change from beamlet intensities to leaf positions, particularly as the impact of changing the latter is often emulated by adapting corresponding beamlet weights.^41^

## 5 CONCLUSIONS

This work introduces a novel scenario‐free robust planning method. It collapses error scenario information into precomputed probabilistic quantities to become independent of the number of scenarios (and partly the number of voxels) during optimization. This way, scenario‐free optimization achieves solid robustness goals, while minimizing memory requirements and optimization time. As a byproduct, the approach facilitates selective reduction of uncertainty within given structures by introducing specific variance reduction cost‐functions, giving direct control over computational complexity as well the robustness of individual structures.

The scenario‐free method was applied to several configurations. These involved a box‐shaped phantom for validation as well as 3D and 4D robust optimization setups on lung patients. The conducted analysis highlighted how this approach allows computationally and memory efficient optimization of robust treatment plans for IMRT and IMPT. The quality and robustness of such plans can compete with traditional optimization strategies, while the optimization time observed for the scenario‐free approach is severely reduced with respect to the traditional robust optimization and indeed close to the nominal, non‐robust approach.

The efficiency achievable with this approach could open the path to the introduction of robust optimization to highly runtime‐constrained optimization scenarios, such as adaptive radiotherapy or multi‐criteria planning, and make robust planning more viable and interactive for clinical applications in the future.

## CONFLICT OF INTEREST STATEMENT

The authors declare that they have no known competing financial interests or personal relationships that could have appeared to influence the work reported in this paper.
